# Perspectives of Adolescents and Young Adults, Caregivers, and Health Care Providers on Regional Cancer Care: Qualitative Study

**DOI:** 10.2196/85096

**Published:** 2026-02-26

**Authors:** Joanne Tay, Mohammad Jarrar, Telford Yeung, Jessica C Kichler

**Affiliations:** 1 Faculty of Nursing University of Windsor Windsor, ON Canada; 2 Department of Pediatrics Windsor Regional Hospital Windsor, ON Canada; 3 Department of Pediatrics University of Calgary Calgary, AB Canada; 4 Department of Psychology University of Windsor Windsor, ON Canada

**Keywords:** adolescent, health services accessibility, health services, neoplasm, qualitative research

## Abstract

**Background:**

Adolescents and young adults with cancer have distinct developmental and psychosocial needs that require care models bridging pediatric and adult oncology systems. While survival outcomes have improved, there is growing recognition of the need to strengthen coordinated care, psychosocial support, and survivorship services. In Ontario, regional and community-based cancer programs play a central role in delivering accessible care and are well-positioned to support adolescents and young adults closer to home. However, variation in infrastructure, workforce capacity, and system-level coordination influences how developmentally appropriate oncology care is implemented. Existing literature has focused on tertiary and specialty centers, highlighting the need to leverage and strengthen regional systems, providers, and community resources to support high-quality adolescent and young adult cancer care.

**Objective:**

This study aimed to explore how structural barriers and supports shape the experiences of adolescents and young adults with cancer, their caregivers, and health care providers (HCPs) in accessing and navigating regional cancer care in Ontario.

**Methods:**

An exploratory qualitative study was conducted between July 2024 and March 2025, guided by Bronfenbrenner’s Ecological Systems Theory and a social constructionist orientation. Semistructured interviews were conducted with 14 participants (adolescents and young adults, caregivers, and HCPs) in Southwestern Ontario. Participants were recruited through purposive sampling. Data were analyzed using reflexive thematic analysis. Trustworthiness and rigor were strengthened through reflexive team discussions and audit trail documentation.

**Results:**

A total of 2 adolescents and young adults, 6 caregivers, and 6 HCPs were interviewed. Youth were in their early 20s and represented different cancer diagnoses. Most caregivers (n=5) were mothers, and HCPs had 2-20 years of experience. Six key themes were generated: (1) structural gaps in coordinated care, (2) reliance on individual champions, (3) developmental and environmental misalignment, (4) barriers to youth engagement and autonomy, (5) gaps in psychosocial, palliative, and survivorship support, and (6) local system enablers. Participants described fragmented care poorly suited to adolescents’ and young adults’ needs. Care environments often defaulted to child- or adult-oriented models, limiting autonomy and engagement. Psychosocial, palliative, and survivorship supports were unevenly available, particularly during transitions off treatment. Despite these challenges, local satellite clinics and community organizations reduced travel burden, fostered continuity, and provided long-term emotional and financial support.

**Conclusions:**

Adolescent and young adult cancer care in regional settings is delivered within evolving systems adapting to youth’s developmental and psychosocial needs. Our findings suggest that targeted investments in workforce development, coordinated communication structures, and age-appropriate care models can further enhance regional capacity to support adolescents and young adults across the cancer trajectory. By shifting from reliance on informal solutions toward more structured, team-based approaches, regional programs can build resilient, youth-responsive systems that promote high-quality, equitable adolescent and young adult cancer care.

**International Registered Report Identifier (IRRID):**

RR2-10.2196/76877

## Introduction

### Adolescents and Young Adults With Cancer: Challenges Within Care

Adolescents and young adults with cancer represent a distinct population whose needs have historically been overlooked within pediatric and adult oncology systems [1]. While overall cancer survival has improved, adolescents and young adults continue to experience slower gains and face persistent inequities in access, supportive care, and survivorship services [1]. Cohort and qualitative studies published over the past several years indicate that these inequities persist despite increased recognition of adolescent and young adult–specific needs, with ongoing gaps in psychosocial support, care coordination, and survivorship planning across cancer systems [2-4]. In Ontario, policy efforts have sought to promote developmentally appropriate care, yet regional and community settings often lack the infrastructure and resources to deliver it [5]. Within this context, adolescents and young adults continue to encounter challenges that extend beyond medical treatment.

Cancer diagnosis during adolescence and young adulthood presents unique challenges extending beyond medical treatment to encompass educational, social, and developmental disruptions [6,7]. Globally, adolescents and young adults with cancer experience structural misalignment between health care systems designed for children or adults and the developmental needs of youth in transition [6,8]. Unlike younger children who rely on parental advocacy or adults who navigate systems independently, adolescents and young adults are in a distinct developmental stage characterized by emerging autonomy, identity formation, and increasing self-determination [9]. However, health care structures are often inadequately designed to recognize this developmental stage, creating what literature has described as a dual disadvantage where youth experience gaps in both pediatric and adult care systems [10]. Despite growing evidence documenting high psychosocial distress, disrupted life trajectories, and unmet developmental needs among adolescents and young adults with cancer, much of this literature remains concentrated in tertiary or specialty care contexts, offering limited insight into how these challenges are navigated within regional and community-based systems [11-14].

### Systemic Barriers Beyond Clinical Care

The structural inadequacy extends beyond clinical care into broader systems surrounding adolescent and young adult cancer treatment. Educational institutions face challenges accommodating youth with fluctuating health needs, employers demonstrate limited understanding of cancer’s long-term impacts, and social support systems frequently default to child-focused or adult-oriented models that inadequately address adolescent developmental needs [3]. The convergence of these structural barriers creates cumulative disadvantages that may persist beyond treatment completion, impacting educational achievement, career development, and psychosocial outcomes [15-17]. Within the Canadian health care context, accessible care delivery is a core mandate for Ontario’s community hospitals [18,19]; yet, achieving equitable access to cancer services between community and tertiary oncology centers remains an ongoing challenge, particularly for adolescents and young adults [12].

### An Ecological Systems Lens

Bronfenbrenner’s Ecological Systems Theory provides a framework for examining how systemic barriers operate across multiple levels to constrain adolescent and young adult cancer care delivery [9]. At the exosystem level, institutional policies, funding structures, and professional training deficits establish structural conditions that either facilitate or constrain quality care delivery. Research applying this framework has documented how institutional constraints, including unclear mandates, underresourced settings, and workforce limitations, compromise the system’s capacity to address youth-specific needs [2,20]. Despite efforts to decentralize tertiary care pediatric oncology services through initiatives such as the Pediatric Oncology Group of Ontario (POGO) and its satellite clinics, substantial community-based inequities in adolescent and young adult care persist. While some satellite sites provide essential follow-up and supportive care for younger patients, adolescents and young adults older than 18 years or those requiring specialized interventions must access tertiary centers for diagnosis, treatment initiation, and subspecialist consultation [16,21]. For youth navigating developmental transition characterized by increasing autonomy, identity formation, and life planning, these structural care disruptions may be particularly destabilizing [22]. This theoretical lens guided our examination of system-level conditions, policies, organizational processes, and service structures, rather than focusing solely on individual experience.

### Theoretical Underpinning

A social constructionist epistemological orientation and relativist ontological stance shaped the study, recognizing that participants’ accounts reflect socially situated interpretations of their interactions with health systems. By incorporating perspectives from youth, caregivers, and health care providers (HCPs), the design enabled an integrative view of the organizational and structural dynamics influencing adolescent and young adult cancer care.

### Resource Constraints in Community Hospitals

In community health care settings, these systemic challenges are amplified by infrastructure limitations and resource constraints. Community hospitals are mandated to deliver outpatient and supportive care; however, many lack the necessary infrastructure, staffing, and specialized services to provide comprehensive, developmentally appropriate adolescent and young adult oncology care [16,23,24]. Consequently, youth and their families in smaller communities require travel, often repeatedly, to tertiary centers for diagnosis, treatment, and acute care management [21,25]. These care disruptions create substantial logistical, financial, and emotional burdens, particularly for families with limited socioeconomic resources, single-parent households, newcomers to Canada, and those with reduced informal support networks [3].

Community hospitals typically operate with limited oncology-specific resources, creating challenges for clinical teams who must adapt adult or pediatric protocols for adolescent and young adult patients without specialized training or institutional support [8]. Care providers often lack training in adolescent and young adult oncology, with limited guidance on managing complex psychosocial issues or coordinating with tertiary care teams [2,20]. Systemic limitations often necessitate reliance on informal champions, typically nurses or allied health professionals, who also function as care coordinators in the absence of more structured support. While their contributions are essential, this reliance on individual practitioners highlights deeper systemic vulnerabilities, including insufficient interdisciplinary teams, the absence of contingency planning, and inadequate infrastructure to support workforce sustainability. High provider turnover, burnout, and service discontinuity represent recurring challenges, particularly in settings where care delivery depends on limited personnel [8,12]. Community hospitals serving adolescents and young adults frequently lack resources, training, and capacity to deliver developmentally appropriate care [7,26]. Youth perspectives are often marginalized in these settings, where reliance on parental involvement in care inadequately addresses developmental needs for autonomy and self-advocacy within an adolescent and young adult population [17].

### Knowledge Gaps in Community-Based Adolescent and Young Adult Oncology

Much of the existing research has examined travel burdens and navigation barriers in tertiary and adult oncology settings, leaving the experiences of adolescents and young adults and their families in community settings comparatively underexplored [26,27]. Care providers in these contexts face considerable challenges in addressing service gaps and supporting youth through complex medical and psychosocial transitions [8,20]. Understanding how systemic barriers operate across multiple ecological levels in community contexts is essential for developing interventions that address the underlying causes of care fragmentation.

### Study Objectives

To explore how structural barriers and supports shape the experiences of adolescents and young adults with cancer, their families, and HCPs in accessing and navigating regional cancer care.

## Methods

### Study Design

We used an exploratory qualitative design informed by Bronfenbrenner’s Ecological Systems Theory [9]. This design was chosen to help investigate and understand adolescents and young adults’, caregivers’, and HCPs’ experiences with oncology care within the complex health care systems. The study is reported in accordance with the Consolidated Criteria for Reporting Qualitative Research.

### Sampling and Recruitment

Recruitment took place between July 2024 and March 2025. Purposive sampling was used to recruit participants who represented diverse social contexts, including variation in gender identity, family composition, and neighborhood socioeconomic background. Eligibility criteria for youth included (1) diagnosis of cancer between January 2018 and December 2024, (2) age 15-18 years at time of diagnosis, (3) residence in Southwestern Ontario and receipt of care through the pediatric oncology satellite clinic, (4) completion of treatment and at least 3 months in remission prior to interview, (5) adequate communicative and cognitive ability to participate, and (6) fluency in English. Youth currently on active treatment, recently diagnosed (<3 months), or identified by clinicians as being at risk of psychological harm from participation were excluded. Parents or caregivers of eligible youth were invited to participate, with English fluency as the only inclusion criterion. HCPs were recruited using the same approach, with study information distributed through community hospital networks and professional contacts, and interested individuals contacting the research team directly. Eligible HCPs included physicians, nurses, social workers, and child life specialists with direct clinical responsibilities in adolescent and young adult cancer care within regional hospitals or POGO satellite clinics. Eligibility was confirmed during initial contact through brief screening questions aligned with the study’s inclusion criteria prior to scheduling interviews. Data analysis was conducted with all participants as a whole rather than as 3 separate groups (adolescents and young adults, caregivers, and HCPs), as each group had fewer than the required 8 participants. Data saturation was met when no new data yielded new insights.

### Data Collection

Recruitment materials (information sheet and poster) were distributed through the POGO satellite clinic in Southwestern Ontario and via closed social media networks, including ChildCan and POGO. Interested individuals responded by contacting the research team directly using the contact information provided on the study materials. This recruitment approach supported purposive sampling by enabling the identification of participants with direct experience of adolescent and young adult cancer care within community hospital settings, including youth, caregivers, and HCPs involved in care delivery. Semistructured interviews were conducted individually by JT or MM based on participants' expressed availability. Two youth who were interviewed were young adults and preferred to be interviewed independently without their caregiver. Microsoft Teams meeting invites were emailed to participants for access to the meeting; contact information was provided if any technical difficulties arose. Verbal consent for study participation and audio recording was obtained during the meeting prior to proceeding with the interview. Consent was treated as an ongoing process, and participants were reminded that they could decline to answer questions or withdraw from the interview at any time without consequence. Interviews were audio recorded on Microsoft Teams and lasted 45-90 minutes, after which they were transcribed and verified for accuracy prior to analysis. Additional procedural details are available in the published protocol [28].

Youth interviews focused on their daily lives and routine, along with their transition throughout treatment. Caregiver interviews revolved around the emotional impact of navigating the health care system and experiences with caregiving. HCP interviews focused on care delivery, navigating systemic barriers, and recommendations for care. See Multimedia Appendices 1 and 2 for interview guides.

### Data Analysis

Analysis followed Braun and Clarke’s [29] 6-phase reflexive thematic analysis, which supported the identification of recurring patterns while attending to the contextual meanings expressed by participants. Transcripts were deidentified and managed in NVivo (version 15; Lumivero) to help organize and manage codes and find patterns for theme development. The Bronfenbrenner’s Ecological System framework was used to help with data analysis and theme development to structure themes around adolescent and young adult oncology care within the complex and multifaceted layers of the health care system [9].

In Phase 1, team members individually reviewed transcripts (JT, MM, and BG) to identify features related to systemic processes, access pathways, and coordination challenges. In Phase 2, data were coded with attention to organizational and structural dynamics, rather than individual coping or emotional responses. Initial codes were inductively developed through iterative engagement with the data, involving the application and refinement of descriptive labels to meaningful text segments and constant comparison across transcripts. For example, segments describing repeated travel to tertiary centers due to unavailable local services were initially coded as “care fragmentation” and later integrated into a broader theme reflecting systemic discontinuity in regional adolescent and young adult cancer care. In Phases 3-5 (theme generation, review, and refinement), codes were iteratively developed into potential themes and reviewed across youth, caregiver, and HCP accounts to ensure coherence and depth at the system level. As part of this process, data from youth, caregivers, and HCPs were examined both within and across participant groups to identify areas of convergence, complementarity, and divergence in experiences of regional adolescent and young adult cancer care. The final phase synthesized themes into a thematic structure that reflects how system organization, developmental alignment, and community-level conditions influence adolescent and young adult cancer care experiences. Both inductive and deductive reasoning informed analysis, drawing on existing adolescent and young adult oncology models to refine interpretation.

Early coding differentiated between individual experiences and system-level processes. Through team discussion, codes related to travel burden, delayed communication, and unavailable services were grouped as structural issues rather than individual concerns, reflecting their shared system-level origins. Themes were retained based on their presence across youth, caregiver, and HCPs’ accounts, rather than their frequency within a single group, to support relevance across roles and settings. Divergent cases were examined to refine themes and ensure the findings reflected variation in regional adolescent and young adult cancer care experiences rather than a single dominant perspective. Any disagreements were discussed until a consensus was reached. Regular team debriefings supported critical reflection on emerging patterns and facilitated discussions across the diverse participant groups, including youth, caregivers, and HCPs.

### Ethical Considerations

This study received ethical clearance from the Windsor Regional Hospital Research Ethics Board (REB 24-477) and the University of Windsor Research Ethics Board (REB 24-027). All participants were English-speaking and resided or worked in the Windsor-Essex region. Informed consent was received prior to proceeding with the interview and audio recording. Both interviewers, a registered pediatric nurse and a research assistant trained in qualitative interviews, are trained in detecting signs of distress and pausing interviews if needed. Mental health resources were also provided to participants during interviews. Interviews were then transcribed verbatim by trained research assistants, deidentified, and stored on a secure computer. Participants were then given an electronic gift card via email.

### Rigor and Reflexivity

To strengthen the rigor and trustworthiness of our findings, we used several strategies, including methodological alignment, role-based triangulation, maintaining an audit trail, and ongoing reflexive practice. Three research team members (JT, MM, and BG), each with qualitative research experience, collaboratively led the coding and thematic development, bringing complementary perspectives to the analysis. We systematically recorded analytic decisions and individual reflections in an audit trail, revisiting these notes and consulting with the research team to ensure interpretations remained closely tied to the data. To enhance credibility and transferability, the research team documented and discussed key analytic decisions throughout the analysis. Throughout the study, we remained reflexive by journaling and openly examining how our disciplinary training and varying clinical involvement in adolescent and young adult oncology shaped our analytic lens and helped mitigate potential biases. This reflexive process helped us foreground participant voices and mitigate the influence of preexisting assumptions.

## Results

### Participant Characteristics

A total of 16 participants signed up for the study, but 2 did not meet the eligibility criteria. As a result, 14 participants were interviewed, including 2 youth, 6 caregivers, and 6 HCPs. Among the caregivers, the majority (5/6) were mothers, with 1 father. Youth participants were in their early 20s and represented different cancer diagnoses. HCPs included professionals with varied roles, such as child life specialist, family support liaison, pediatrician, pharmacist, and registered nurses. There was a range of 2 to >20 years of experience in adolescent and young adult oncology among HCPs. Full demographic details are provided in Table 1.

**Table 1:** Participant characteristics.

Variable						Value		
**Parents (n=6)**								
	Age in years, mean (range)				47 (39-53)			
	**Ethnicity, n (%)**							
		White			5 (83.3)			
		Prefer not to answer			1 (16.7)			
	**Education, n (%)**							
		High school diploma			1 (16.7)			
		College/trade school			2 (33.3)			
		University			1 (16.7)			
		Postgraduate degree			1 (16.7)			
		Other			1 (16.7)			
	**Employment status, n (%)**							
		Not employed			1 (16.7)			
		Part-time			2 (33.3)			
		Full-time			3 (50)			
	**Relationship to youth who has cancer, n (%)**							
		Mother			5 (83.3)			
		Father			1 (16.7)			
**Youth (n=2)**								
	Age in years, mean (range)			23 (22-24)				
	**Ethnicity, n (%)**							
		White	2 (100)					
	**Education, n (%)**							
		High school diploma	1 (50)					
		College/trade school	1 (50)					
	**Employment status, n (%)**							
		Not employed	1 (50)					
		Full-time	1 (50)					
**Health care providers (n=6)**								
	**Length of time providing oncology care to young adults and youth, n (%)**							
		<5 years	2 (33.3)					
		5-10 years	1 (16.7)					
		>10 years	3 (50)					
	Number of young adults and youth in current practice, mean (range)			14 (5-25)				

### Overview of Themes

#### Thematic Summary

Analysis generated 6 interconnected themes that illuminate how structural barriers operate across ecological system levels to constrain adolescent and young adult cancer care. These themes demonstrate the complex relationship between individual experiences and systemic deficiencies that characterize regional adolescent and young adult oncology. These themes are (1) structural gaps in coordinated and equitable oncology care; (2) overreliance on individual champions in an underresourced system; (3) developmental and environmental misalignment in youth cancer care; (4) barriers to youth engagement, autonomy, and voice; (5) gaps in psychosocial, palliative, and survivorship supports; and (6) system enablers that support youth and families. Findings reflect converging and contrasting perspectives across youth, caregivers, and HCPs, highlighting shared challenges as well as role-specific experiences within regional adolescent and young adult cancer care.

#### Structural Gaps in Coordinated and Equitable Oncology Care

HCPs consistently mentioned fragmented communication and uneven care quality between tertiary and regional centers as key barriers to coordinated adolescent and young adult oncology care. Despite the need for youth-centered care, participants described a disconnect across sites, leaving both HCPs and caregivers underinformed and youth vulnerable to suboptimal care.

Structural gaps in coordinated and equitable oncology care were described as multifaceted and interconnected. Across participant groups, these gaps encompassed fragmented communication between tertiary and regional centers, uneven access to clinical expertise and oncology-trained personnel, and limited availability of medications and treatment protocols in community settings. Communication breakdowns included inconsistent written documentation, delayed information sharing, and limited direct access to tertiary specialists, leaving regional teams and families to bridge gaps informally. Together, these structural deficiencies undermined continuity, timely decision-making, and eroded confidence in the coordination of care across settings, particularly within regional follow-up and shared care pathways, even as confidence in tertiary oncology expertise remained high. One regional HCP articulated this disconnection vividly:

[The youths] don’t tell me anything. They will go to their tertiary care centre and then divulge all this information to their oncologist, but that sometimes doesn’t get back to me.

HCP01

These communication gaps hindered the delivery of timely and responsive care, placing added pressure on families who were left to navigate disconnected systems on their own. Providers described encountering patients during follow-up appointments without access to critical discharge information, leading to delays and uncertainty:

Patients come back for follow-up, and it is not in the discharge doctor’s note. I don’t know what I am supposed to do. Patients have to wait until we figure it out ... there are a lot of barriers there and parents get upset.

HCP04

Rather than functioning as part of an integrated care system, regional teams were often left reactive, informed only when issues arose:

There’s room for improvement in terms of more consistent check-ins and streamlined communication between the care teams in [tertiary center] and [regional center]. Communication usually occurs only when there’s an issue.

HCP06

These accounts illustrate how fragmented communication extended beyond interpersonal exchanges to include gaps in written discharge documentation, delayed information transfer, and limited direct communication channels between regional providers and tertiary oncology teams. While adolescents and young adults and families continued to seek and trust tertiary oncologist expertise, confidence in the system’s ability to function as a coordinated whole was diminished, especially during transitions and regional follow-up.

Beyond communication, participants described structural gaps related to limited clinical expertise and staffing capacity in regional settings, which constrained the ability to deliver consistent, oncology-informed care. Parents, particularly those with clinical backgrounds, described regional teams as underresourced and underprepared for the demands of pediatric oncology:

We need trained, more educated nurses who know what they’re doing with respect to oncology pediatric patients ... if there was none on the floor, they had to call the adult oncology nurse to come.

P06

From the provider perspective, limited caseloads and infrastructure posed ongoing challenges to maintaining expertise:

You want experts in the field who are seeing this daily. So, if you can’t support that with volume ... the care is watered down.

HCP06

Structural inequities were also evident in access to medications and treatment protocols, with families encountering limitations to locally available therapies and inconsistent access to chemotherapy regimens. These constraints often required families to travel to tertiary centers or accept suboptimal symptom management in regional settings. One parent described the frustration of discovering that their regional center would not carry a nausea medication that had been effective for their child:

There was a medication which they figured out for my son’s nausea [but] our cancer center does not have it ... they will not carry it or even order it for you.

P06

Others described having to travel to a tertiary treatment center to access specific chemotherapy not available at the local satellite clinic:

He could get some [chemo] here at the satellite clinic, but there were certain kinds that ... he’d have to travel to [tertiary treatment center] for the day and then come back.

P05

Continuity of care was frequently sustained through the consistent engagement and relationship-building efforts of individual providers. Their ongoing presence created stability for families within care pathways that were not always seamlessly connected, highlighting that relational continuity often compensated for, rather than resolved, underlying structural fragmentation, and underscoring opportunities to strengthen system-level structures that can better support this work.

#### Overreliance on Individual Champions in an Underresourced System

Youth cancer care in smaller cities was often sustained by a few dedicated champions, who served as the relational and procedural anchors in the absence of coordinated infrastructure. These longstanding relationships became vital for families navigating a complex and emotionally charged care journey. Nurses who had followed youth through multiple treatment cycles were often seen as irreplaceable. One parent reflected on the central role of a trusted nurse:

[The nurse] is someone who is crucial for [the cancer care in the city] ... [she] was a very big part in like, I guess the good part of the cancer diagnosis ... Built a relationship with [youth] as well, so that was a familiar face.

P04

Similarly, a provider described the unique role of a long-serving staff member who became the “pillar” of the clinic:

I will say that our [satellite] clinic is fantastic in terms of our recent lead staff, who had just retired. I think she was ... the pillar of the clinic. And so, I can definitely appreciate her longitudinal relationships with these children, and you know that consistency and comfort that she brought to the clinic.

HCP06

Another provider described the relational disconnect that arose when covering for a colleague on leave:

They trust the [healthcare provider] very much, and I have a harder time bonding with that family. I always try really hard to get the parents to open up about how they’re feeling.

HCP04

While the relational model provided meaningful continuity of care, it placed considerable strain on those stepping into these “individual champion” roles temporarily. Relief staff were expected to handle not only complex clinical tasks such as port access and chemotherapy preparation, but also to meet the relational expectations usually fulfilled by familiar providers. The same provider further reflected on this challenge, noting, “when it’s not that primary person, it is difficult and very nerve-wracking for me” (HCP04), highlighting the implicit comparison to a trusted key team member. Participants described how high parental expectations and the lack of team-based backup intensified provider stress:

Parents were constantly hovering and commenting, ‘will the staff be able to do it like [nurse] does?’ ... and I was stressed. There are not enough people who are confident with ports ... I did not have a backup.

HCP05

This fragility in having trusting relationships when receiving regional cancer care was not limited to nursing. Other HCPs also reported similar vulnerabilities due to understaffing and role overload. In contrast to tertiary centers, which had designated personnel for oncology services, regional providers often worked in isolation across multiple service areas:

Tertiary center has two [healthcare providers of the same role] just dedicated to oncology. I am doing all of these alone ... How do we make it so that it’s not just one person?

HCP05

While these individual efforts were essential to maintaining continuity, they also underscored the extent to which the care experience relied on personal relationships rather than systemic design. Familiarity with a trusted provider was a powerful buffer against an environment that otherwise felt disjointed from the needs of youth. Without systemic structures in place to ensure developmental responsiveness, care often defaulted to rigid categories (ie, child or adult) that failed to reflect the developmental realities of adolescents and young adults. This disconnect surfaced not only in staffing and coordination but also in the broader environments where care and recovery unfolded.

#### Developmental and Environmental Misalignment in Youth Cancer Care

Youth, parents, and providers consistently described both regional and tertiary cancer care environments as misaligned with the developmental and psychosocial needs of adolescents and young adults. Designed mainly for children or adults, current systems often leave adolescents and young adults without appropriate spaces or supports, reinforcing feelings of isolation, exclusion, and disempowerment. Many participants described health care environments that were either child-centric or adult-oriented, with no age-appropriate options in between. A provider noted, “We have a pediatric or an adult cancer ward. But what about the young adults?” (HCP05). This forced placement into inappropriate programs often made adolescents and young adults feel out of place and visibly different. One parent recalled the discomfort of being placed in an adult support group:

They had registered us for the adult [cosmetic] program ... and so we were in a room full of women ... and my daughter felt very uncomfortable.

P02

Beyond issues of age-appropriateness, participants also pointed to systemic limitations in accommodating youth under immunosuppressive protocols. One youth described the social isolation of being physically present but barred from meaningful interaction:

Even when I did go back, I wasn’t allowed to go around people. I was just able to see them from a distance.

Y01

Even when youth were physically present, the surrounding structures seldom provided space for independent voice, reflection, or choice, deepening their sense of invisibility within the system.

This disconnect within exosystems extended beyond health care into education systems as well, where youth and families encountered similar structural gaps in communication and developmental support. For some, physical inaccessibility in school environments compounded the recovery process and emotional toll:

Her mobility was hugely restricted ... and I found that the school was not very understanding ... didn’t provide extra time ... loaded her up with heavy textbooks.

P02

Educational disruption and poor reintegration into school activities were among the most frequently raised concerns. Youth described being met with apathy or misunderstanding from teachers, contributing to a sense of abandonment at a time when support was most needed:

It’d be nice just for [teachers] to be a little more supportive, not just kind of shun you or shove you off.

Y01

Parents echoed these frustrations, noting the absence of structured communication between hospital-based care and educational institutions. In their view, the lack of coordinated roles and clear processes left families to navigate these transitions on their own:

It would be nice to have more collaboration between the contact person at the hospital and the schools ... There wasn’t really a process or a system of support in place that I found was really adequate.

P06

Even when educational support was available, it was often not tailored to the developmental stage of older youth. As one provider explained, volunteer tutoring programs, while well-intentioned, frequently fell short of meeting the academic needs of senior high school students:

We have a volunteer tutoring program ... but for kids that are in grades 11 and 12... It’s very difficult to find someone to help who is qualified.

HCP02

Youth also described the emotional burden of having to repeatedly explain their cancer history to peers and teachers, an experience that underscored the lack of systemic communication mechanisms:

It would be nice if the school knew [so] I didn’t have to restate everything and relive all the things that happened.

Y01

These mismatches between care environments and developmental needs not only left youth feeling out of place in schools and hospitals but also reinforced patterns of exclusion that limited their ability to participate meaningfully in their own care.

#### Barriers to Youth Engagement, Autonomy, and Voice

As mentioned earlier, adolescence is a crucial period for developing autonomy and identity. Yet, youth, parents, and providers described barriers to engaging adolescents and young adults as active participants in their care. Young people often felt sidelined by the need for constant parental presence in a hospital setting and health care systems that lacked the structure to center the youth voices in decision-making. The most immediate barrier was the structural absence of private, youth-directed spaces in clinical settings. Providers noted that youth rarely had opportunities to speak independently, as parents were nearly always present during appointments:

When they’re in the clinic or admitted to the hospital, there is always a parent present. They don’t have any time or opportunity to express themselves without a parent listening.

HCP01

Even when youth were physically present, the surrounding environment often lacked opportunities for independent voice, reflection, or choice, which may have contributed to a sense of invisibility within the system. In these situations, clinicians observed that parents often took the lead in communication, sometimes speaking on behalf of the youth.

You know, it was ... trying to ask them if they had any questions ... kind of engage them in their care versus their parents ... [instead] I get the parents’ answer.

HCP05

These missed opportunities for confidential conversations with clinicians were seen as limiting not only for youth but also for providers trying to build rapport:

[Private spaces or time alone with youths] would be better at helping them open up ... but parents are always there with the kids

HCP02

The consequences of these missed youth-provider interpersonal interactions were twofold. First, youth often disengaged entirely, either due to discomfort or lack of perceived agency:

With the youth and adolescent population, I struggle ... I feel like I don’t necessarily always have the time to explore all the underlying issues ... It’s more superficial.

HCP06

Second, parents often became the sole conduit for emotional disclosures, an arrangement that left providers relying on second-hand reports to assess psychosocial needs. One provider noted:

The biggest issue is that [youth] don’t talk about their feelings; parents will usually reach out to me.

HCP02

This not only limited the ability to engage youth authentically but also risked reinforcing dependency at a time when adolescents are developmentally primed to begin asserting independence and autonomy. Providers emphasized the importance of structured, developmentally appropriate approaches to foster youth voice and participation:

As they’re trying to navigate adolescence and personal identity ... young adult transition clinics would be quite important ... they need to build that trust before being able to actually open up.

HCP06

Some pointed to peer-facilitated spaces as promising platforms for agency and shared learning:

There needs to be a peer-on-peer family group ... having a facilitator could ensure accuracy and support.

HCP04

Yet even when youth sought to engage, the absence of integrated psychosocial, palliative, and survivorship supports left many of their broader needs unmet.

#### Gaps in Psychosocial, Palliative, and Survivorship Supports

The psychosocial aspects of youth cancer care, especially mental health, end-of-life support, and survivorship systems, were described as fragmented, underresourced, and overlooked. Although essential for long-term well-being, these supports are often less developed and less available than medical services. Mental health emerged as a particular area of concern. Parents described the cumulative emotional trauma usually experienced by their children and families and lamented the lack of proactive mental health resources or referrals.

There’s a lot of trauma. And for no sort of mental health support to be recommended for you to get referrals, no one coming in to talk about it, that is very concerning ... Not just for the child, but for parents, caregivers, and siblings too.

P06

Access to services was also marked by inequities. Providers and parents pointed to long waitlists for youth counseling and the financial barriers to accessing private care. One provider and one parent noted:

I don’t really think that [mental health] support is widely accessible. There are long waitlists for accessing mental health services, and going private depends on whether they have the money or benefits.

HCP02

I think if there was counselling later that was offered, that wasn’t a cost or whatever ... She definitely does have some PTSD [post-traumatic stress disorder] ... and OHIP [Ontario Health Insurance Plan] should cover counselling for critical conditions.

P05

For many, the systemic gaps in support became particularly stark during the survivorship and transition periods for the youth. Parents and HCPs both noted that psychosocial support tended to decrease once treatment ended:

You’re 17, and everything is organized. At 18, you’re expected to navigate it all on your own ... Youth transition clinics would be quite important. Smaller cities would definitely benefit from that.

HCP06

It almost seems like there needs to be a period of transition into adult care ... not just jump from a tertiary children’s centre to the adult oncology.

P03

This abrupt transition was especially difficult for youth experiencing developmental vulnerability:

The transition wasn’t gradual; it was sudden. It was becoming stressful for me because he didn’t belong there. He belongs around more fun things. Not the adult cancer centre.

P02

Equally concerning were the significant gaps in palliative care and bereavement support. Families reported being left to navigate end-of-life planning and grief without structured guidance or psychological care:

Try to imagine planning a funeral and a birthday party three days apart ... There is absolutely nothing there [to support a family member after death]... You are so alone.

P02

Even when community programs existed, they were often underfunded or unavailable to many:

The grief program for kids through CMHA [Canadian Mental Health Association] has no additional funding ... It’s purely [based] on fundraising.

P06

The challenge of finding trauma-informed therapy was ongoing and deeply taxing for some parents.

All the trauma he went through and all the nightmares. Thank God, we took him for therapy ... but it was not easy to find.

P02

While many families faced challenging moments in the cancer journey without adequate psychosocial or palliative support systems, their experiences also revealed what mattered when care felt responsive. Participants highlighted local, community-based, and satellite-driven supports that eased the burdens of treatment and recovery.

The [satellite] clinic was very helpful ... for blood work or the chemo he could get here instead of us having to travel to [tertiary treatment center].

P04

It was really good to be close to home, because I could go home and rest after ... And [nurse name] was just always there ... It wasn’t some new person every time. It made it feel like people cared, like I wasn’t just a number.

Y01

These were not incidental benefits but crucial system enablers that, when present, enhanced access, continuity, and emotional well-being for youth and their families.

#### System Enablers That Support Youth and Families

While participants acknowledged systemic gaps in cancer care, they also highlighted key supports that eased the burden for youth and families. Local satellite clinics, financial assistance, and community-based resources provided critical support. Access to nearby care reduced travel strain, though participants emphasized that proximity must be matched by adequate staffing and resources to ensure safe, high-quality care.

It would be nice to have more care closer to home, provided we could, if we had the resources to do it safely and the necessary support staff. We are a satellite center, and we must acknowledge that we are not the primary one, but it would be nice.

HCP05

Youth also voiced a desire for greater local capacity, envisioning a future where advanced therapies, like stem cell transplants, might eventually be delivered in regional settings.

More local treatment on a grander scale, more options locally to do more treatment here, even so far as like the stem cell transplants.

Y02

Alongside local access, financial and transportation support were key. For many families, unexpected levels of financial assistance, offered through local charities and hospital partnerships, helped offset the high costs of treatment, travel, and time away from work. As one parent shared: “I was very surprised by how much financial support there really is” (P03). Providers also noted that these systemic supports extended beyond the treatment period, with some organizations offering educational bursaries to youth survivors pursuing postsecondary education: “[Community organization] gives bursaries for postsecondary students.” (HCP01).

The long-term engagement model offered by community organizations like ChildCan and Fight Like Mason was especially valued [30]. These groups were not seen as peripheral supports, but as integral components in the care ecosystem, offering emotional, logistical, and financial assistance that extended well beyond hospital walls. Youth and families described these relationships as enduring, often continuing for years after active treatment had ended. One provider noted: “Even ten years later, [survivors] can apply for a bursary” (HCP02). This long-term community support was especially valuable in smaller cities, where formal survivorship and mental health services were limited. Community organizations provided a sense of continuity and connection when formal systems withdrew, ensuring that families did not feel abandoned at the end of treatment.

These accounts highlight the importance of flexible, locally embedded supports that bridge gaps in formal care. While satellite clinics, financial aid, and community organizations may not replace the comprehensive health care infrastructure of tertiary centers, they serve as crucial scaffolding, helping youth and families stay connected, supported, and hopeful throughout and beyond the cancer care journey.

## Discussion

### Principal Findings

This study explored how adolescents and young adults with cancer, their caregivers, and HCPs experienced regional cancer care in Ontario, and identified key barriers to developmental alignment, care coordination, and equitable access within these systems. Our findings reveal structural gaps that create challenges for delivering equitable care to adolescents and young adults with cancer. Communication between tertiary and regional centers is inconsistent, leaving families to rely on individual champions rather than reliable systems. Fragmented communication had implications beyond information exchange, shaping relationships, trust, and continuity of care. Inadequate or delayed discharge documentation limited direct communication channels, and inconsistent access to oncology specialists left regional providers uncertain about care plans and families unsure whom to rely on for guidance. These gaps shifted the burden of coordination onto caregivers and strained therapeutic relationships, particularly for nurses who were expected to provide continuity without access to complete or timely information. From a nursing perspective, such breakdowns undermine relational care by constraining providers’ ability to anticipate needs, follow through on care plans, and build confidence with youth and families. These findings highlight the need for more formalized, standardized communication processes between tertiary and regional teams to support continuity, accountability, and relationship-centered care in adolescent and young adult oncology. Care environments often default to binary pediatric or adult models, with adolescent and young adult autonomy and psychosocial needs frequently unaddressed. Gaps in mental health, palliative care, and survivorship services were also evident throughout the care journey, reflecting broader misalignments within existing systems. In this context, families described drawing on local supports and community organizations outside of the formal care system. Our findings point to an opportunity to better align existing communication processes with the needs of adolescent and young adult oncology care across institutional boundaries, drawing on structured handover approaches already used in other areas of health care.

In regional settings, individual providers often took on responsibilities beyond their formal roles to deliver adolescent and young adult cancer care. While this is consistent with rural oncology research indicating that committed professionals sustain care continuity [8,31], our findings suggest that more systematic infrastructure could help strengthen these efforts. This pattern may reflect broader challenges, including infrastructure gaps, insufficient system-level guidance, and limited provider education in developmentally appropriate adolescent and young adult care [8,32]. Reliance on key individuals rather than a sustainable infrastructure means that a single provider’s departure can disrupt service continuity and erode trust among patients and families [33,34]. While countries like the United Kingdom and Australia have made strides toward centralized adolescent and young adult care frameworks, implementation remains inconsistent across many jurisdictions, including Canada, leaving individual champions to fill systematic gaps [8,20]. This has implications for workforce sustainability and system resilience, as documented challenges arise when providers operate beyond their scope without adequate support [35-38]. Our findings suggest that while individual champions remain important and should be celebrated, there is significant potential to evolve toward well-resourced, team-based models that ensure more consistent, equitable adolescent and young adult cancer care [6,20]. These dedicated efforts could be enhanced through more systematic support to improve adolescent and young adult care quality and access across regional settings.

Another key insight from our study is the mismatch between existing care models and the developmental realities of adolescents and young adults. Pediatric care often centers on parental involvement, which can delay the development of independence, while adult care assumes emotional and cognitive maturity that many adolescents and young adults are still developing [2,4,39]. Such gaps contribute to disengagement and disruptions in care continuity. The misalignment is further complicated when adolescents and young adults are treated in spaces that do not foster trust or reflect their evolving identities [12,15]. Without youth-appropriate space and support, many are reluctant to raise sensitive issues or participate fully in care decisions [10]. These gaps extend beyond clinical care into important life domains, with our participants describing educational interruptions, limited attention to fertility, and a lack of structured support for work reintegration. These patterns echo the BRIGHTLIGHT cohort findings of high psychosocial distress among adolescents and young adults due to ineffective communication and insufficient age-appropriate guidance [17], highlighting the need for developmentally relevant supports in sexuality, education, and career planning often overlooked in adult oncology settings [32].

Psychosocial, palliative, and survivorship care remains inconsistently delivered in cancer care settings despite growing awareness of their importance [17,26]. Our findings demonstrate that regional variation in care quality and access creates equity barriers even in publicly funded systems like Canada [27,40,41]. Extending this work, our study also highlights how these gaps are experienced within regional and community-based settings, where limited mental health infrastructure and workforce shortages further constrain access to counselling, bereavement, and trauma-informed care, and our study participants described feeling abandoned during care transitions and struggling with unresolved trauma and grief without access to counselling or bereavement support [20,31,38]. These gaps were particularly significant in rural areas, where mental health infrastructure and workforce shortages were limited [12,25,31]. Psychosocial supports are often overlooked as biomedical treatment takes priority over emotional recovery and life-stage reintegration, partly because of gaps in integrating trauma-informed psychosocial care due to inadequate funding and lack of structural support [10,32]. As a result, families are left to navigate fragmented supports independently, adding emotional strain and worsening inequities.

### Strengths and Limitations

This study is limited by its focus on 1 region (Southwest Ontario), which may affect transferability to other health care contexts. A key strength of this study lies in the integration of perspectives across youth, caregivers, and HCPs, enabling examination of converging and contrasting accounts of regional adolescent and young adult cancer care. This sample is predominantly White, which may affect generalization to other populations. While purposive sampling was used to ensure variation across key social characteristics, such as age, socioeconomic background, and geographical location, it may not fully capture diversity across other rural and urban regions in Canada. Although the study included 2 adolescents and young adults with different diagnoses, the sample size is limited, which may be due to the study’s specificity and inclusion criteria. This may not reflect the perspectives of youth with other oncology diagnoses or those entirely disengaged from cancer care, such as those lost to follow-up or experiencing ongoing structural exclusion. Due to resource constraints, only parents or caregivers with English fluency were included within the study, which can limit the diversity of this sample.

### Comparison With Prior Work

Much of the existing literature has focused on broad national or international perspectives, including the Institute of Medicine workshop summary [10] and the *Canadian Partnership Against Cancer’s Adolescents and Young Adults with Cancer: A Reference Report* [12]. Our study offers a regional perspective on how these systemic issues manifest in day-to-day cancer care delivery. While large-scale cohort investigations such as BRIGHTLIGHT emphasize psychosocial outcomes [17] and qualitative studies of advanced disease foreground existential concerns [11], our analysis focuses on how structural barriers and developmental misalignments are experienced within a smaller, underserved Canadian region. This regional perspective complements existing models-of-care reviews [20,32] by illustrating how resource limitations and workforce reliance shape the care landscape in ways not fully captured in national or tertiary-level studies.

### Recommendations for Further Research

Future research should explore findings across broader geographic contexts, including interprovincial comparisons of adolescent and young adult oncology models, and examine how structural determinants, such as income, Indigeneity, and gender, may intersect with access to cancer care and subsequent outcomes. Mixed method studies integrating patient-reported outcomes, service use data, and system-level indicators could also deepen understanding of how developmental misalignment and fragmentation affect quality, equity, and survivorship trajectories. Longitudinal research is needed to assess the effectiveness of targeted system interventions, such as youth navigation roles or regional adolescent and young adult coordinators.

### Implications for Policy and Practice

To advance equity and quality in adolescent and young adult oncology, policy and practice may benefit from prioritizing care models that reflect young people’s developmental contexts, including age-appropriate education, counseling, and clinical environments that support autonomy. Although models such as age-specific care settings and coordinated transition services have shown promise, their implementation has been uneven and often limited to well-resourced settings [6,20]. Sustained improvements will likely require investment in cross-institutional coordination, provider training, and supportive policies, with examples showing that dedicated funding and leadership can drive progress [8,20].

Within this study, nurses were consistently identified as central to supporting adolescents and young adults, often taking on coordinating and advocacy roles despite limited formal recognition or specialized training. These findings suggest opportunities for strengthening nursing contributions in adolescent and young adult care, such as (1) integrating developmental psychology into nursing education, (2) establishing advanced practice adolescent and young adult care coordinator roles, (3) fostering youth-friendly care environments, (4) incorporating trauma-informed approaches to address mental health needs, and (5) developing adolescent and young adult–specific interventions through nursing-led research. Advancing these areas will require institutional support, including dedicated funding, protected coordination time, and recognition of nursing expertise, to move from informal efforts toward more structured, developmentally responsive systems of care.

### Conclusion

Adolescents and young adults with cancer receiving care in regional settings often face services that are fragmented, not fully aligned with developmental needs, and unevenly supported across systems. Families described depending on the efforts of individual clinicians, particularly nurses, to bridge service gaps, while psychosocial, palliative, and survivorship supports were less consistently available. Local clinics and community organizations played an important role in providing accessible and ongoing support, particularly close to home. These findings suggest a need to transition from individually driven solutions to more reliable, coordinated, and developmentally responsive models of adolescent and young adult cancer care that integrate equity, sustainability, and youth voice.

## Data Availability

The datasets generated during or analyzed during this study are available from the corresponding author on reasonable request.

## Appendix

Multimedia Appendix 1 Interview guide for health care providers.

Multimedia Appendix 2 Interview guide for youth and parents/caregivers.

## Abbreviations

HCP: health care provider
POGO: Pediatric Oncology Group of Ontario
