# Supplementary material for: Perspectives of Adolescents and Young Adults, Caregivers, and Health Care Providers on Regional Cancer Care: Qualitative Study
Source: JMIR Cancer. 2026 Feb 26;12:e85096. doi: 10.2196/85096 (PMC12982960; doi:10.2196/85096)
Supplement: Multimedia Appendix 1 [file cancer_v12i1e85096_app1.docx]

**Qualitative Interview Guide for Healthcare Providers**

1. Tell me a little bit about yourself and your role as a [healthcare provider e.g., oncologist, dietician, registered nurse, occupational therapist etc.] in the Oncology Satellite Unit?
2. What experience do you have providing oncology care specifically to the adolescent and young adult population?

**Probes:**

- - What is your relationship like with the youths and their families?
  - Please elaborate some of the challenging moments you have with the youths and their families when providing care.
  - What did you hope to have that might make these challenging moments easier?

1. The availability of timely and appropriate support is an important aspect in oncology care for youths and their families. Can you describe some of the community and hospital/clinic resources that are available for youths living with cancer and families in Windsor?

**Probes:**

- - Why do you think these resources are/are not useful for the youths?
  - Are there resources that you think should be made available to youths and their families but do not currently exist?
  - Why do you think these resources are important specifically for youths?
  - How do you think resources should be tailored for youths and their families?
  - What should be included in these resources?

1. What do you think are some of the important considerations that youths have in mind when undergoing their cancer treatment?

**Probes:**

- - Please elaborate some of the things that youths and their families discuss with you before, during, and after their cancer treatment.
  - Tell me a little bit about discussions you had with the youths, if any, about other concerns other than their medical ones e.g., pursuing higher education, career, family planning, marriage etc.
  - What do you think might be helpful for you as a healthcare provider to navigate these personal questions with the youths and their families?

1. How do you think cancer care in the Oncology Satellite Unit at Windsor Regional Hospital can be improved for youths with cancer?

**Probes:**

- - What are some of the gaps that you think exist in services and care provided to youths with cancer?
  - What are some of the ways that these gaps can be addressed?

1. Is there anything else you feel that we did not cover today or that you feel is important for me to know about this topic? Do you have any questions for me?
