# Supplementary material for: Perspectives of Adolescents and Young Adults, Caregivers, and Health Care Providers on Regional Cancer Care: Qualitative Study
Source: JMIR Cancer. 2026 Feb 26;12:e85096. doi: 10.2196/85096 (PMC12982960; doi:10.2196/85096)
Supplement: Multimedia Appendix 2 [file cancer_v12i1e85096_app2.docx]

**Qualitative Interview Guide for Youths and Parents/Caregivers**

1. Tell me a little bit about yourself and your family?
2. What has it been like for you/your child to live with cancer?

**Probes:**

- - What was it like for you/your child to be diagnosed?
  - How has the experience of you/your child being diagnosed with cancer impacted you?
  - What challenges did you/your child encountered when receiving oncology care at Windsor Regional Hospital? (e.g., clinical care/interactions with your healthcare provider, psychosocial support specifically for youths)
  - What were some of the supports you/your child received that helped you/your child in your/their cancer journey in Windsor? Why did you think those were helpful?

1. Tell me about the resources that you used or were referred to during your cancer treatment journey? In what ways did you find them helpful or not helpful?

**Probes:**

- - Please elaborate if these resources met your needs.
  - How have these supports addressed your concerns relating to your peers and school?
  - Did these resources consider your long term needs and wants?
  - In what ways do you think this support might help you to make future decisions on issues such as higher education, career, family planning, marriage etc.

1. Some youths tell us that cancer is a difficult and isolating process. They find it hard to even talk to their child/parents about how they are feeling. How have your conversations with your parents/child been like?

**Probes:**

- - What type of supports did you receive from your parents during your cancer treatment?
  - How did you feel your parents could have better supported you in your journey?

1. Finding a timely and appropriate support is an important aspect in oncology care for youths. I am going to ask you some questions about the supports you have used or are still using.

**Probes:**

- - Tell me a bit about the support you received from your healthcare providers. In what way were this support able to meet your needs.
  - Tell me some of the community supports that you used during and after your cancer treatment. How do you think these supports were relevant in addressing your needs?

1. Tell me a bit about what is most important to you in life right now?

**Probes:**

- How do you think your healthcare providers (e.g., doctors, nurses, and others) could have better prepared you or inform you about the challenges you will face during your/your child’s treatment and life after treatment?

1. What do you hope to see improved in terms of cancer care for youths living in Windsor?

**Probes:**

- - What are some of the gaps that you think exist in services and care provided to youths with cancer?
  - What are some of the ways that these gaps can be addressed?

1. Is there anything else you feel that we did not cover today or that you feel is important for me to know about this topic? Do you have any questions for me?
